# Climate Vulnerability and Human Migration in Global Perspective

**DOI:** 10.3390/su9050720

**Published:** 2017-04-30

**Authors:** Martina Grecequet, Jack DeWaard, Jessica J. Hellmann, Guy J. Abel

**Affiliations:** 1 Institute on the Environment, University of Minnesota, St. Paul, MN 55108, USA; 2 Department of Sociology and Minnesota Population Center, University of Minnesota, Minneapolis, MN 55455, USA; 3 Department of Ecology, Evolution and Behavior, College of Biological Sciences, University of Minnesota, St. Paul, MN 55108, USA; 4 Asian Demographic Research Institute, School of Sociology and Political Science, Shanghai University, Shanghai 200444, China; 5 Wittgenstein Centre for Demography and Global Human Capital (IIASA, VID/ÖAW, WU), Vienna Institute of Demography (Austrian Academy of Sciences), Vienna 1020, Austria

**Keywords:** climate change, climate vulnerability, international migration, migration flows, life-supporting sectors, ecosystem services

## Abstract

The relationship between climate change and human migration is not homogenous and depends critically on the differential vulnerability of population and places. If places and populations are not vulnerable, or susceptible, to climate change, then the climate–migration relationship may not materialize. The key to understanding and, from a policy perspective, planning for whether and how climate change will impact future migration patterns is therefore knowledge of the link between climate vulnerability and migration. However, beyond specific case studies, little is known about this association in global perspective. We therefore provide a descriptive, country-level portrait of this relationship. We show that the negative association between climate vulnerability and international migration holds only for countries least vulnerable to climate change, which suggests the potential for trapped populations in more vulnerable countries. However, when analyzed separately by life supporting sector (food, water, health, ecosystem services, human habitat, and infrastructure) and vulnerability dimension (exposure, sensitivity, and adaptive capacity), we detect evidence of a relationship among more, but not the most, vulnerable countries. The bilateral (i.e., country-to-country) migration show that, on average, people move from countries of higher vulnerability to lower vulnerability, reducing global risk by 15%. This finding is consistent with the idea that migration is a climate adaptation strategy. Still, ~6% of bilateral migration is maladaptive with respect to climate change, with some movement toward countries with greater climate change vulnerability.

## 1. Introduction

Climate change is already altering weather patterns and ecological processes in ways that are consequential for human populations [1]. Human migration is one such example that is increasingly important in scholarly and policy circles. Migration is an adaptive strategy—one of many and often one of last resort—used to mitigate livelihood threats, including those due to climate change [2,3].

Many international initiatives now recognize the link between climate change and migration, one that will likely grow more pronounced given the decline of ecosystem services, increasing constraints on natural resources, and associated socioeconomic and geopolitical pressures under climate change [4–6]. At the 21st Conference of Parties (COP 21) under the United Nations Framework Convention on Climate Change (UNFCCC), the Executive Committee requested the formation of a task force to make recommendations on how to address climate-related displacement [7]. Migration is also a part of the 2030 Agenda for Sustainable Development [8]. A follow up to the Nansen Initiative, the Platform on Disaster Displacement likewise addresses the needs of cross-border displaced persons [9]. The Sendai Framework for Disaster Risk Reduction explicitly recognizes population displacement and planned relocation as key policy issues [10].

Given growing recognition of the association between climate change and migration, it is important to recognize that this relationship is strongly heterogeneous [11]. The climate-migration relationship depends on the differential vulnerability of places and populations to climate change, which, in turn, is a function of their unique exposure, sensitivity, and adaptive capacity [12,13]. As such, there is no necessary relationship between climate change and migration. Accordingly, the key to determining whether and how climate change will impact future migration patterns is a better understanding of the association between climate vulnerability and migration. Presently, however, there is a dearth of research on this association, most especially at the global level [11,14].

In this paper, we examine the association between climate vulnerability and international migration in 179 countries. First, we explore the spatial pattering of countries’ climate vulnerability scores in 2010 and rates of net-migration during the 2010–2015 period. Second, we analyze the association between climate vulnerability scores and rates of net-migration going back to the mid-1990s. Third, given heterogeneity in the association between climate vulnerability scores and rates of net migration, we disaggregate our analysis by climate vulnerability quartile and life supporting sectors. Finally, shifting from rates of net migration to bilateral (i.e., country-to-country) migration flows, we consider whether and to what extent migration flows between and within vulnerability quartiles exhibit evidence of a gradient whereby migration is directed from more to less vulnerable countries.

## 2. Data

### 2.1. Climate Vulnerability Scores

Country-level data on climate vulnerability are drawn from the Country Index of the Global Adaptation Initiative at the University of Notre Dame (ND-GAIN) (Available online at http://index.gain.org/) [15]. The Country Index is an established metric used by scholars and policy makers (e.g., the Green Climate Fund and the World Economic Forum, among others) to study climate risk and adaption opportunities [16]. Presently, the Country Index has not been used in studies of migration.

The Country Index defines vulnerability as the propensity or predisposition of human societies to be negatively impacted by climate hazards [1]. Climate vulnerability scores in the Country Index are constructed from 36 indicators across six life supporting sectors (food, water, health, ecosystem services, human habitat, and infrastructure) tapping three dimensions of vulnerability (exposure, sensitivity, and adaptive capacity) (Supplementary Table S1).

Exposure refers to changes in biophysical factors that affect human society and its supporting sectors (changes in crop yields, marine biodiversity, etc.). Sensitivity refers to the degree to which human society and its supporting sectors are affected by climate disturbances, with sensitivity indicators including, for example, countries’ dependency on climate-sensitive sectors (e.g., agriculture) and the proportion of the population that is sensitive to climate hazards due topography (e.g., living in low-lying coastal areas). Adaptive capacity refers to the ability to respond to the negative consequences of climate change, with indicators of adaptive capacity serving as proxies of possible actions that may ameliorate the impacts of climate change (fertilizer and pesticide use, access to electricity, area of protected biomes, engagement in international environmental conventions, etc.).

We note that vulnerability scores in the Country Index are negatively correlated with per capita Gross National Income (GNI) [16], which is consistent with the broad consensus that economic growth and development contributes to vulnerability reduction. However, while wealthier countries may have more resources at their disposal to adapt to climate change, economic growth and development are not the only factors determining vulnerability to climate change. Social and geopolitical factors also play important roles. Vulnerability likewise varies across life supporting sectors.

Indicators in the Country Index are scaled (normalized) to range between zero and one, and then aggregated by (un-weighted) averaging within sectors. Countries’ climate vulnerability scores can therefore be interpreted relative to one another. Climate vulnerability scores are available annually from 1995 to 2015. We used climate vulnerability scores for the years 1995, 2000, 2005, and 2010, as these four years correspond to the first years of the four observation windows in our migration data.

### 2.2. International Migration Flows

Country-level data on migration include a newly developed set of estimates of bilateral (i.e., country-to-country) flows [17,18]. For each pair of countries worldwide, these take the form of five-year counts of the minimum number of persons who migrated between each pair of countries, which were estimated via a likelihood procedure that used information from the United Nations on foreign-born population stocks disaggregated by country of birth in national censuses, as well as information on fertility and mortality. These estimates are available for five year periods starting in 1990, 1995, 2000, 2005, 2010, and 2015. To account for changes in geopolitical boundaries over time, we combined migration data for Serbia and Montenegro, as well as for Sudan and South Sudan. We did the same for these countries’ climate vulnerability scores in the Country Index by averaging them. For a given country, the net migration rate is calculated as the difference between total in- and total out-migration flows, divided by total person-years lived in the five-year window. The calculation of person-years used population data from the United Nations [19] and assumed that population growth was linear in the five-year interval.

We used a suite of descriptive and exploratory statistics, including the Moran’s *I* to assess spatial autocorrelation in our measures of climate vulnerability and net migration, the Intraclass Correlation Coefficient (*ICC*) to determine whether and to what extent variation in our measures is due to differences between versus within countries, and the Spearman correlation coefficient (*r_s_*) to summarize the direction and strength of the relationship between our measures.

## 3. Results

### 3.1. Spatial Patterning in Climate Vulnerability and Net Migration

**Figure 1:**
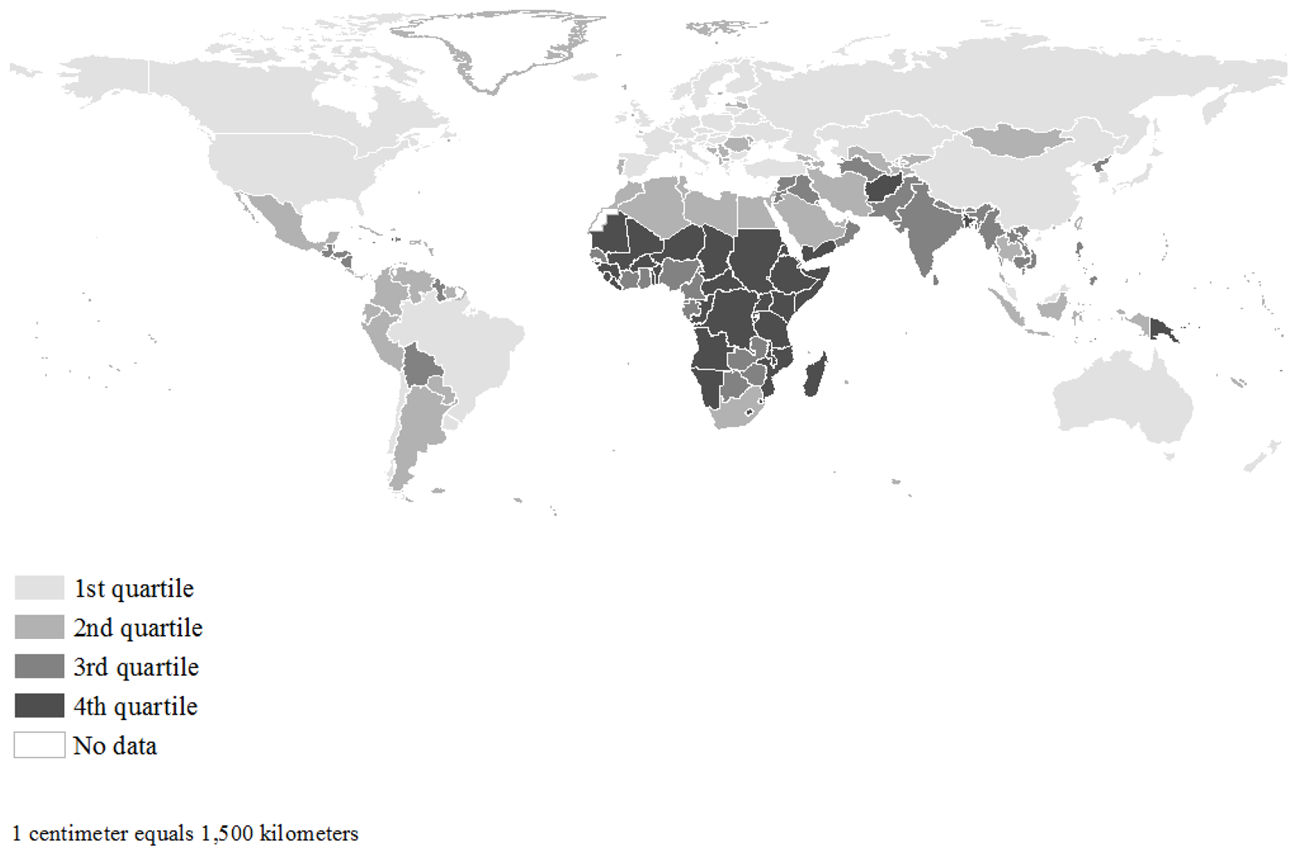
Climate vulnerability score, 2010. Shading reflects climate vulnerability quartiles, with cut points of 0.35 (25th percentile), 0.43 (50th percentile), and 0.54 (75th percentile). Mean climate vulnerability score was 0.44, with a range of 0.47 (min = 0.22; max = 0.69). Data taken from the Country Index of the Notre Dame Global Adaptation Index (ND-GAIN), and cover 179 countries. Data deficient countries shown in white.

There is a clear spatial patterning in climate vulnerability scores across countries in 2010 (Figure 1). Countries in the first climate vulnerability quartile are the least vulnerable to climate change. The majority of the countries in the first climate vulnerability quartile (i.e., least vulnerable to climate change) are located in North America, Europe, and Eastern Asia. Countries in the fourth climate vulnerability quartile are the most vulnerable to climate change, and include most countries in Sub-Saharan Africa, as well as others in South America (e.g., Guyana), Southeastern Asia (e.g., Afghanistan and Bangladesh), and Melanesia (e.g., Papua New Guinea). The Moran’s *I* is positive and is statistically significant (*I* = 0.252, *p* < 0.05), meaning that countries with high (or low) climate vulnerability scores tend to neighbor those with similarly high (or low) climate vulnerability scores. There are, however, clear exceptions to this clustering (Guyana, Papua New Guinea, etc.), such that some of the most vulnerable countries neighbor less vulnerable countries. Similar patterning is observed when climate vulnerability scores are broken down by each dimension of vulnerability: exposure, sensitivity, and adaptive capacity (Supplementary Figures S1–S3).

**Figure 2:**
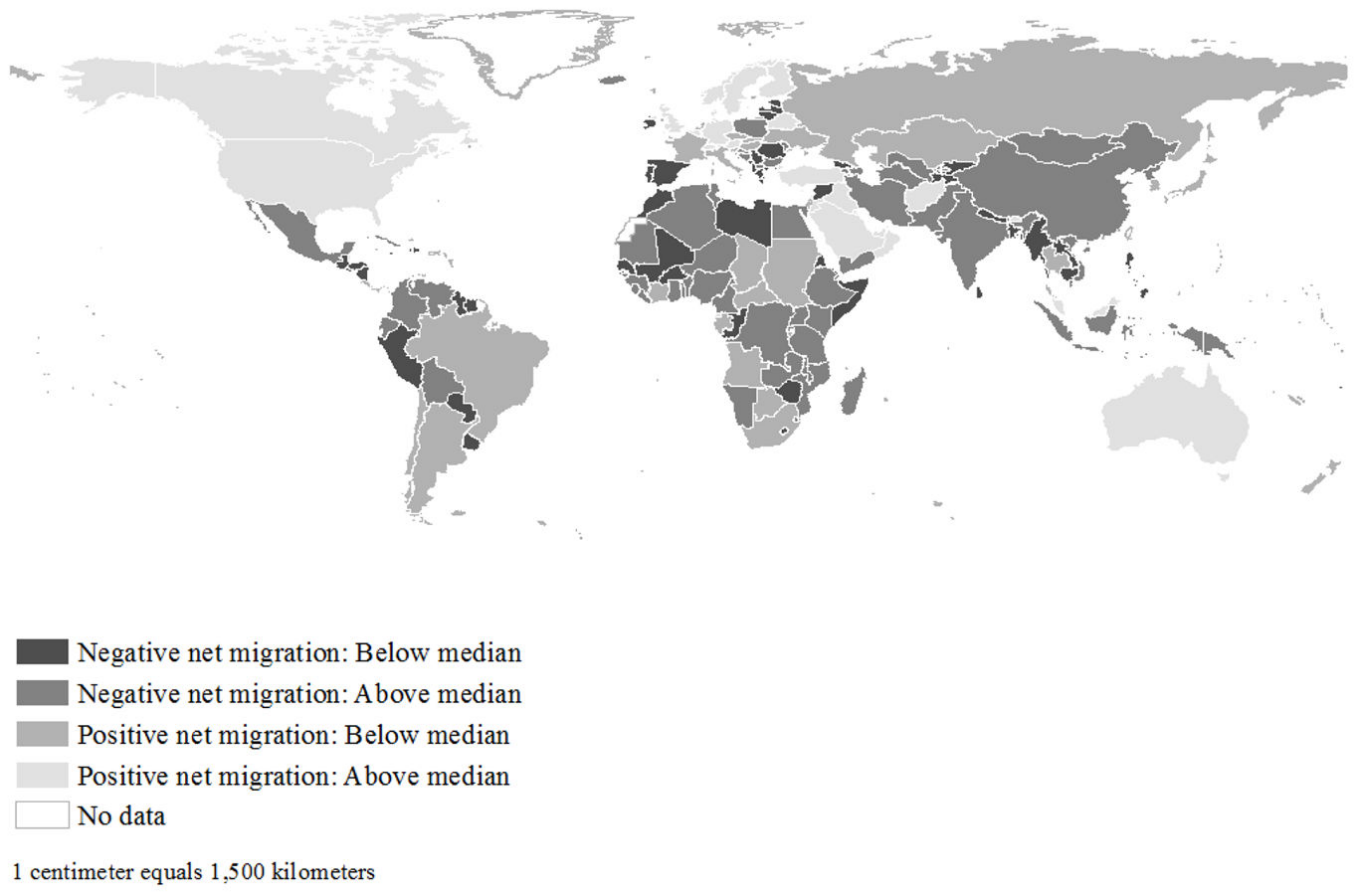
Net migration rate per thousand population, 2010–2015. Net migration rate is an occurrence-exposure rate, calculated as the difference between in- and out-migration flows, divided by total person-years lived in the five-year window. Negative (darker shading) and positive (lighter shading) net migration rates indicate population loss and gain due to migration, respectively. Negative and positive net migration rates are further cut at 50th percentiles, with values of −1.46 and 2.45 per thousand, respectively. Mean net-migration rate was 0.44 per thousand, with a range of 121.18 (min = −38.90 per thousand; max = 82.28 per thousand). Data provided by Abel (2015). Countries for which for which data are not available and/or for which data are not also available from the Country Index of the Notre Dame Global Adaptation Index (ND-GAIN) shown in white.

There also is evidence of spatial patterning in rates of net migration across countries during the 2010–2015 period (Figure 2). Countries with the highest positive rates of net migration experienced the largest population increases due to migration. These include countries in North America (e.g., Canada and the United States), Europe (e.g., Germany, the United Kingdom, and the four Nordic countries of Denmark, Finland, Norway, and Sweden, among others), Western Asia (e.g., Qatar, Saudi Arabia, Turkey, etc.), Oceania (e.g., Australia), and South-Eastern Asia (e.g., Malaysia). Countries with the most negative rates of net migration experienced the largest population losses due to migration. These countries are scattered across most world regions, with Syria, Libya, Tonga, Georgia, and Samoa experiencing the lowest negative rates of net migration. The Moran’s *I* is negative and is statistically significant (*I* = −0.090, *p* < 0.05), meaning that countries with high (or low) net migration rates tend to neighbor those with similarly low (or high) net migration rates. The magnitude of Moran’s *I*, however, indicates weak spatial clustering overall. There are likewise notable exceptions to this pattern in North America, Europe, and Western Asia.

### 3.2. Association Between Climate Vulnerability and Net Migration

**Figure 3:**
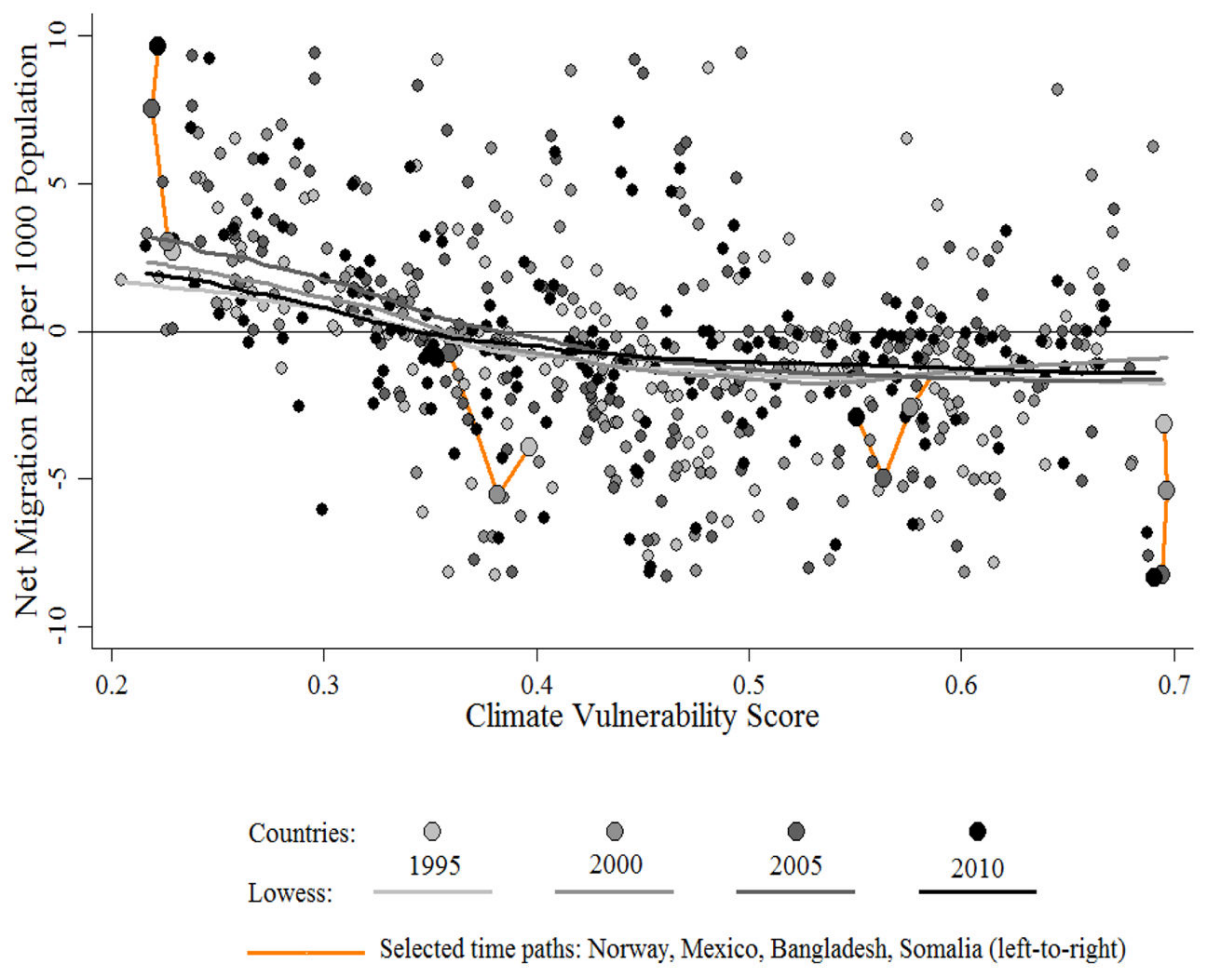
Climate vulnerability score and net migration rate: 1995, 2000, 2005, 2010. With respect to net migration, dates refer to the first year of the respective migration interval (e.g., 1995 refers to 1995–2000). Greyscale circles and lines correspond to countries and lowess plots, respectively. Orange lines correspond to time paths for selected countries between 1995 and 2010. From left-to-right, these countries include Norway, Mexico, Bangladesh, and Somalia.

Over the past two decades, climate vulnerability scores have decreased, with the majority of variation due to changes within, rather than differences between, countries (*ICC* = 0.989, *p* < 0.05). In contrast, rates of net migration have increased (i.e., negative and positive net migration have become less and more so, respectively), with the bulk of variation likewise due to changes within countries (*ICC* = 0.635, *p* < 0.05). However, the association between climate vulnerability and net migration has changed very little over the past two decades, and largely reflects differences between, versus changes within, countries (Figure 3).

Spearman correlations range from *r_s_* = −0.347 (*p* < 0.001) in 2000 to *r_s_* = −0.428 (*p* < 0.001) in 2005. In the most recent period, this association was likewise negative and nonlinear (*r_s_* = −0.356, *p* < 0.001). Changes over time in this association within countries (e.g., in Norway, Mexico, Bangladesh, and Somalia in Figure 3) are generally small and, with one exception, are not statistically significant. The lone exception was between 2005 and 2010, where the correlation between the change in climate vulnerability scores and the change in rates of net migration within countries was *r_s_* = −0.206 (*p* = 0.011). Accordingly, the remainder of this section is focused on between-country variation in the association between climate vulnerability scores in 2010 and rates of net migration during the 2010–2015 period.

**Figure 4:**
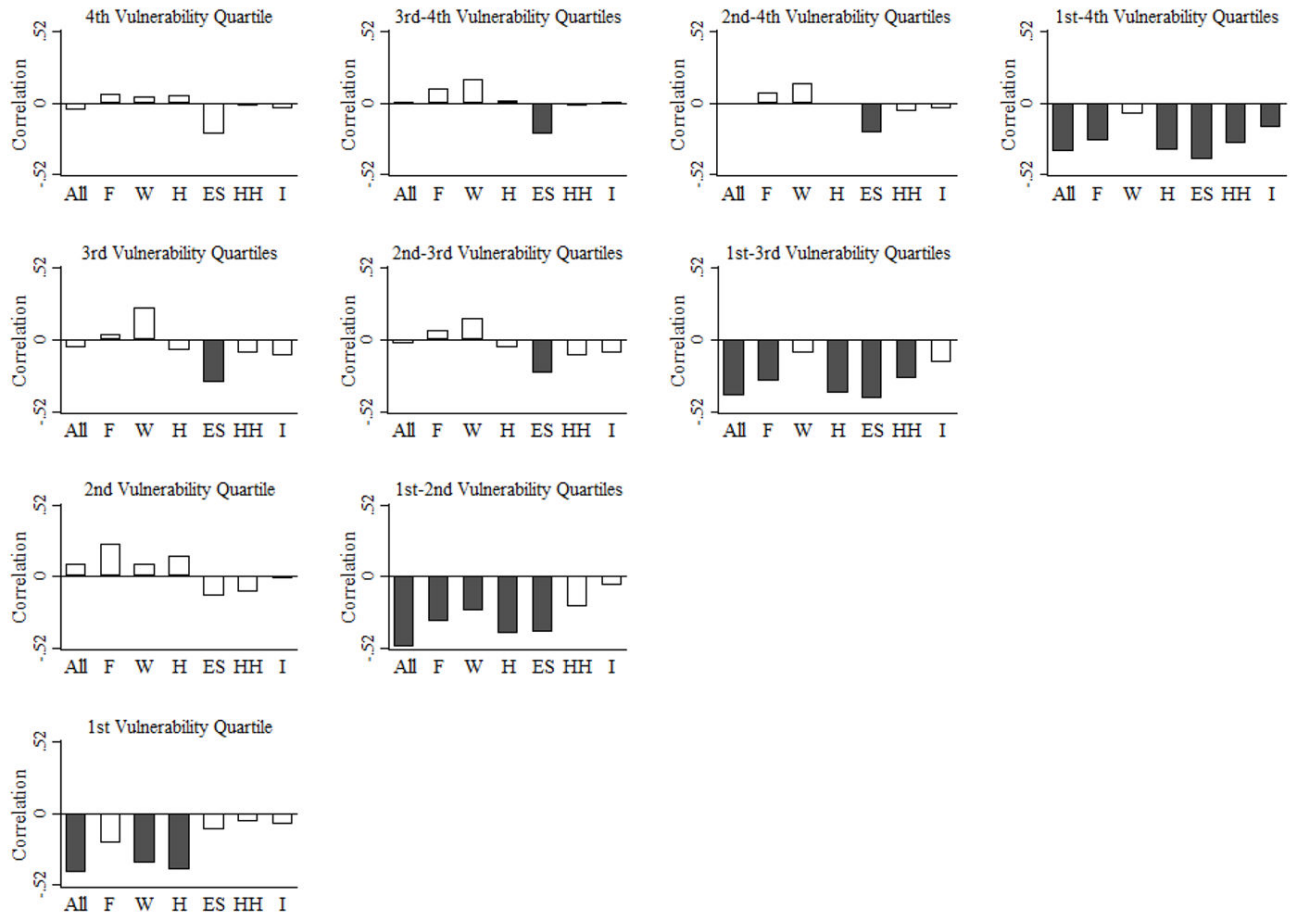
Correlations between climate vulnerability score and net migration rate by life supporting sector and climate vulnerability quartile: 2010. With respect to net migration, 2010 refers to the 2010–2015 period. Sectors include all sectors (All), food (F), water (W), health (H), ecosystem services (ES), human habitat (HH), and infrastructure (I). Shading denotes Spearman correlation coefficient is statistically significant (*p* < 0.05).

Observed nonlinearity in the association between climate vulnerability scores and rates of net migration raises the prospect of trapped populations in countries that are more/most vulnerable to climate change [14,20]. Trapped populations are likely those most vulnerable to climate change (e.g., those locked in deep and persistent poverty) who lack the resources necessary to adapt by migrating. Among countries most vulnerable to climate change (“Fourth Vulnerability Quartile”), there is no association between climate vulnerability scores and rates of net migration (*r_s_* = −0.048, *p* = 0.768) (Figure 4). This finding holds even when climate vulnerability scores are broken down by six life supporting sectors (food, water, health, ecosystem services, human habitat, and infrastructure) and three dimensions of vulnerability (exposure, sensitivity, and adaptive capacity). While these findings are consistent with narratives and recent concerns about trapped populations [21], other explanations are also possible and cannot be ruled out given our aims and data. For example, one explanation is that those living in countries most vulnerable to climate change use other in situ (in place) adaptation options in lieu of migration. Another explanation is that, because international migration is far less common than internal, or domestic, migration, the former might not be a prominent climate-adaptation strategy in countries most vulnerable to climate change.

In countries that are more (third quartile), but not most (fourth quartile), vulnerable to climate change, a negative and statistically significant association between vulnerability in the area of ecosystem services and net migration is evident (*r_s_* = −0.312, *p* = 0.050). The indicators of vulnerability of ecosystem services in the Country Index (Supplementary Table S1) capture the future impact of climate change on biodiversity (measure of exposure), the degree to which countries are sensitive to losses of natural capital and ecological assets (measure of sensitivity), and the capacity to protect ecosystem and biodiversity under stress (measure of adaptive capacity). Countries in the third quartile (e.g., India and Pakistan) are particularly dependent on climate sensitive sectors (e.g., agriculture and forestry). The negative association between vulnerability in ecosystem services and net migration thus suggests population losses via migration due to the decreasing availability of provisioning ecosystem services that support human livelihoods.

**Figure 5:**
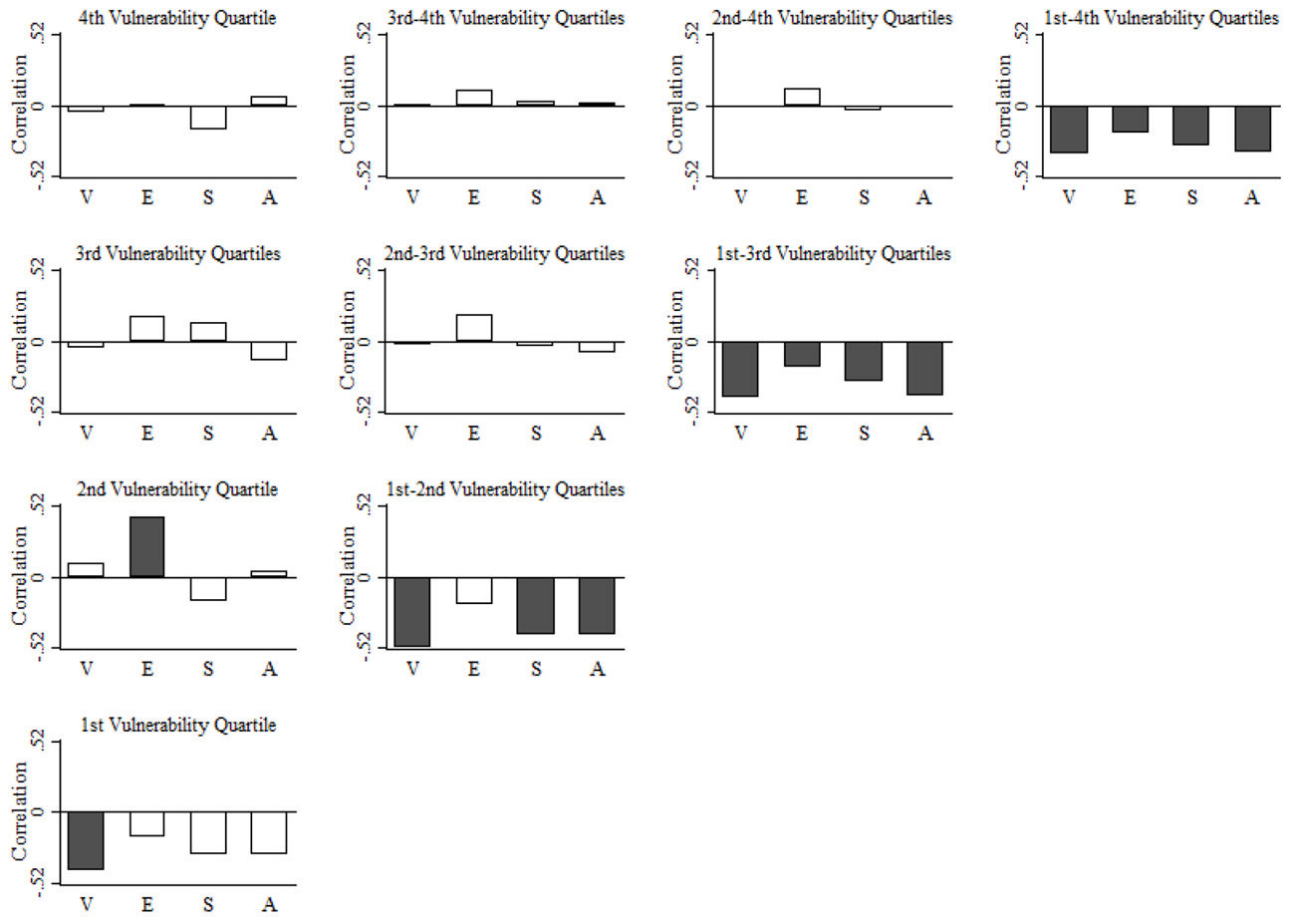
Correlations between climate vulnerability score and net-migration rate by dimension of vulnerability and vulnerability quartile: 2010. Dimensions of vulnerability (V) include exposure (E), sensitivity (S), and adaptive capacity (A). Shading denotes Spearman correlation coefficient is statistically significant (*p* < 0.05).

In countries that are less vulnerable (second quartile) to climate change, there is a positive and statistically significant association between exposure and net migration (*r_s_* = 0.432, *p* = 0.004) (Figure 5). In the Country Index, exposure scores measure the degree to which climate change may affect countries’ life supporting sectors, and are based on series of climate projections [15]. A positive association between exposure and net migration indicates less population loss (in the case of negative net migration) and more population gain (in the case of positive net migration) in areas that may be significantly affected by climate change. These include, for example, countries in West Asia, such as Saudi Arabia or United Arab Emirates that are projected to experience decreases in annual water runoff due to climate change.

### 3.3. Climate Vulnerability and Bilateral Migration Flows

**Figure 6:**
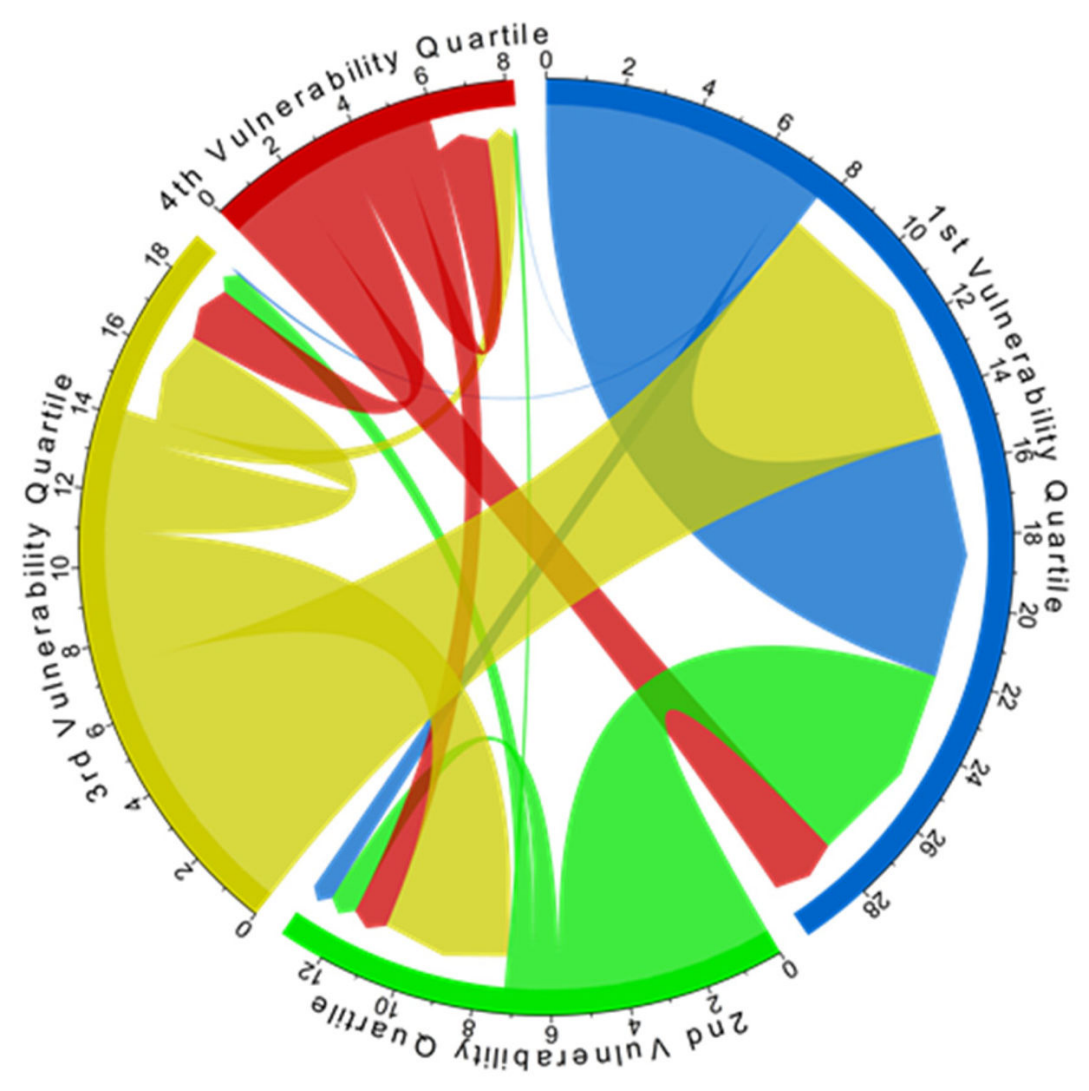
Bilateral migration flows between and within climate vulnerability quartiles: 2010–2015. Colors reflect climate vulnerability quartiles shown earlier in Figure 1. Numbers and tick marks on periphery are counts of out- and in-migrants in units of millions. Migration flows represented by cords. The width of each cord is proportional to the size of the migration flow. The color of each cord denotes migrant-sending (versus migrant-receiving) vulnerability quartile. The arrowhead at the end of each cord further communicates the direction of each migration flow.

Net migration rates (and counts) mask information about the direction of migration flows [22]. This impedes understanding of whether and to what extent migration, as a climate adaptation strategy, follows a vulnerability gradient whereby people migrate from more to less climate vulnerable countries. Focusing on bilateral migration flows (Figure 6), there is clear evidence of a vulnerability gradient. Of the estimated 14.2 million persons who migrated from countries in the third climate vulnerability quartile between 2010 and 2015, 18% migrated to another country in the same quartile, 25% migrated to a country in the second quartile, and 52% migrated to a country in the first quartile. Similar gradients are observed for migration flows from each of the other vulnerability quartiles. The majority of migrants are therefore moving from more to less climate vulnerable countries; however, a non-trivial number (about 6% of all international migrants) moved from less to more vulnerable countries.

## 4. Conclusions

Because the climate-migration relationship is heterogeneous and depends critically on the differential vulnerability of places and populations, it is essential that scholars and policy makers understand the association between climate vulnerability and migration. In less climate vulnerable countries, there is a pronounced negative relationship between climate vulnerability and international migration, with the majority of migration flows directed to less or similarly vulnerable countries. From climate change adaptation perspective, this is positive outcome, because migration helps to decrease the vulnerability of populations to climate change. In contrast, the most climate vulnerable countries are not characterized by pronounced migration, and, in fact, may require substantial aid and targeted interventions to avoid large scale humanitarian emergencies (famine, starvation, etc.) if migration is a not a viable climate adaptation strategy. These associations between climate vulnerability and migration may provide important insights into the future direction and magnitude of migration patterns under climate change. Given that the primary source of variation in the association between climate vulnerability and migration is between (versus within) countries, policy makers must also continue to wrestle with the burden of persistent economic and social inequalities that will only exacerbate large differences in climate vulnerability across countries [23,24].

## Supplementary Material

supplementary
