# Supplementary material for: Climate Vulnerability and Human Migration in Global Perspective
Source: Sustainability. Author manuscript; Available in PMC 2018 May 1. (PMC5912889; doi:10.3390/su9050720)
Supplement: supplementary [file NIHMS958785-supplement-supplementary.pdf]

Sector	Vulnerability			Country Rank (2015)
	Exposure	*Sensitivity*	*Capacity*	
Food	Projected change of cereal yields	Food import dependency	Fertilizer, irrigation, pesticide and tractor use	1.Andorra<br>2. Morocco<br>3. Eritrea
	Projected population change	Rural population	Child malnutrition	
Water	Projected change of annual runoff	Fresh water withdrawal rate	Access to reliable drinking water	1.Monaco<br>2. Macedonia<br>3.San Marino
	Projected change of annual groundwater recharge	Water dependency ratio	Dam capacity	
Health	Projected change of deaths from climate induced diseases	Slum population	Medical staff	1.Australia<br>2.Dominican Republic<br>3.Niger
	Projected change of length of transmission season of vector-borne diseases	Dependency on external resource for health services	Access to improved sanitation facilities.	
Ecosystem Services	Projected change of biome distribution	Dependency on natural capital	Protected biomes	1.Luxenburg<br>2.Monaco<br>3.Tuvalu
	Projected change of marine biodiversity	Ecological footprint	Engagement in International environmental conventions	
Human habitat	Projected change of warm period	Urban concentration	Quality of trade and transport related infrastructure	1.Luxenburg<br>2.Bahrain<br>3.Burundi
	Projected change of flood hazard	Age dependency ratio	Paved roads	
Infrastructure	Projected change of hydropower generation capacity	Dependency on imported energy	Electricity access	1.Andorra<br>2.Macedonia<br>3.Chad
	Projection of Sea Level Rise impacts	Population living under 5 m above the sea	Disaster preparedness	

Table S1. Indicators for vulnerability measures in Country Index of the Global Adaptation Initiative at the University of Notre Dame (ND-GAIN). Includes examples of the (1) least, (2) medium, and (3) the most vulnerable country in each sector based on 2015 ranking.

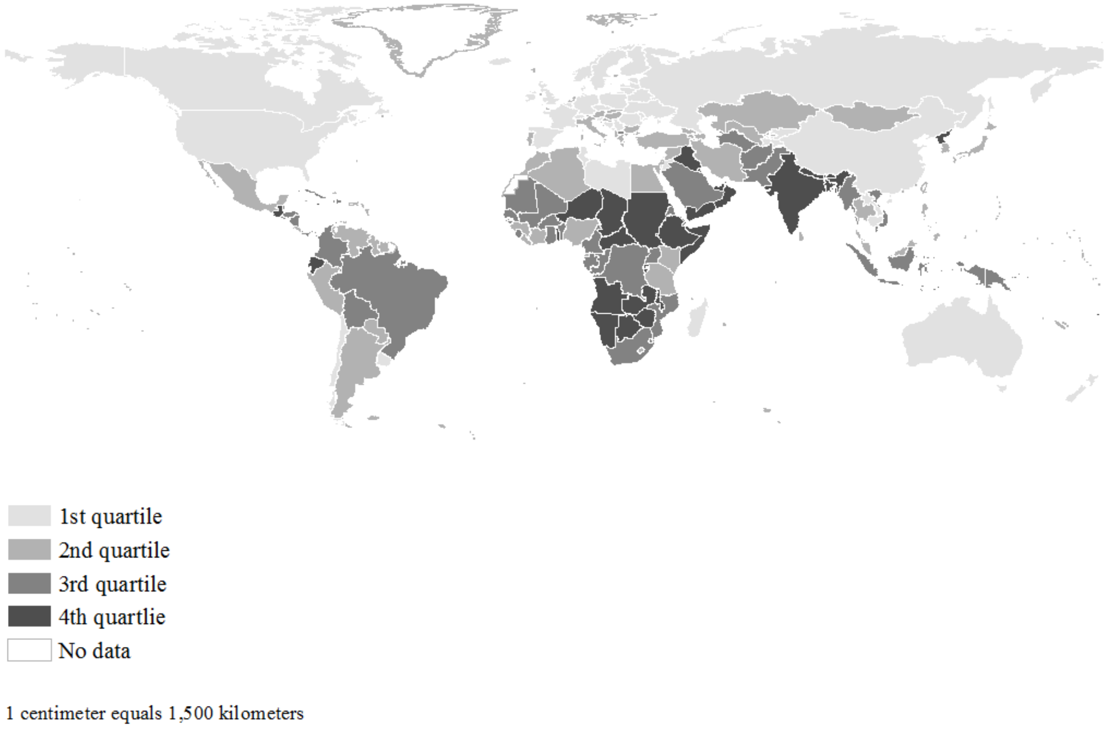

Figure S1 Climate exposure score, 2010. Colors Shading reflects climate vulnerability quartiles, with cut points of 0.43 (25th percentile), 0.49 (50th percentile), and 0.53 (75th percentile). Mean score was 0.48, with a range of 0.42 (min=0.32; max=0.74). Data taken from the Country Index of the Notre Dame Global Adaptation Index (ND-GAIN), and cover 179 countries. Data deficient countries shown in white.

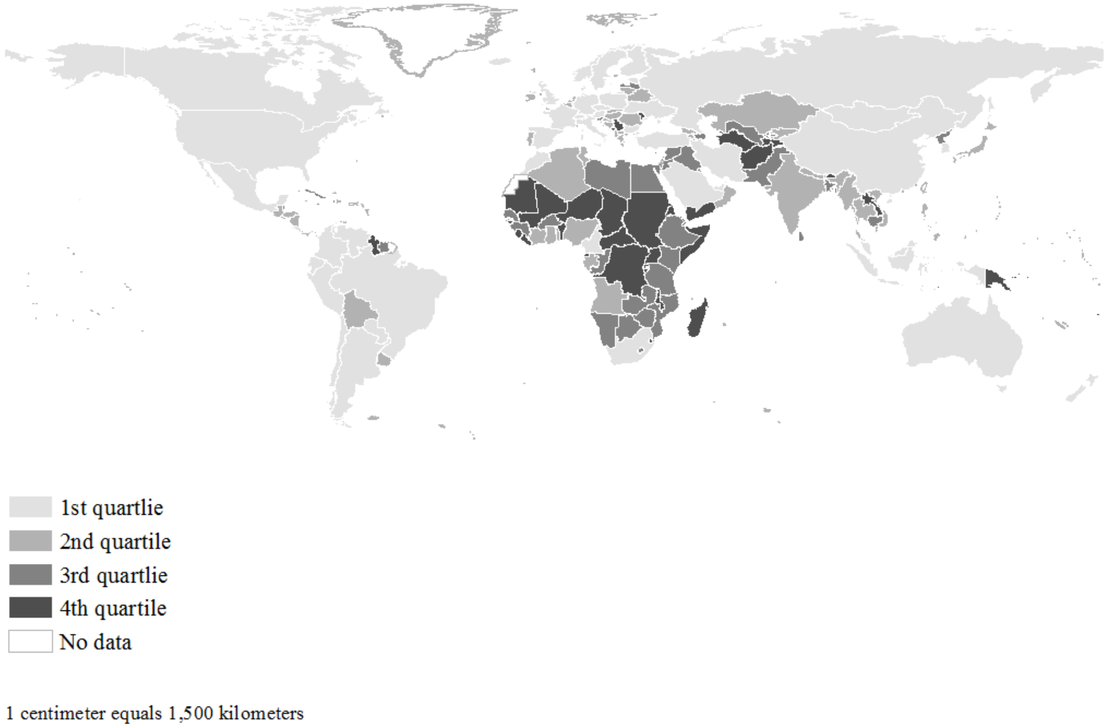

Figure S2 Climate sensitivity score, 2010. Colors Shading reflects climate vulnerability quartiles, with cut points of 0.32 (25th percentile), 0.43 (50th percentile), and 0.52 (75th percentile). Mean score was 0.42, with a range of 0.66 (min=0.14; max=0.80). Data taken from the Country Index of the Notre Dame Global Adaptation Index (ND-GAIN), and cover 179 countries. Data deficient countries shown in white.

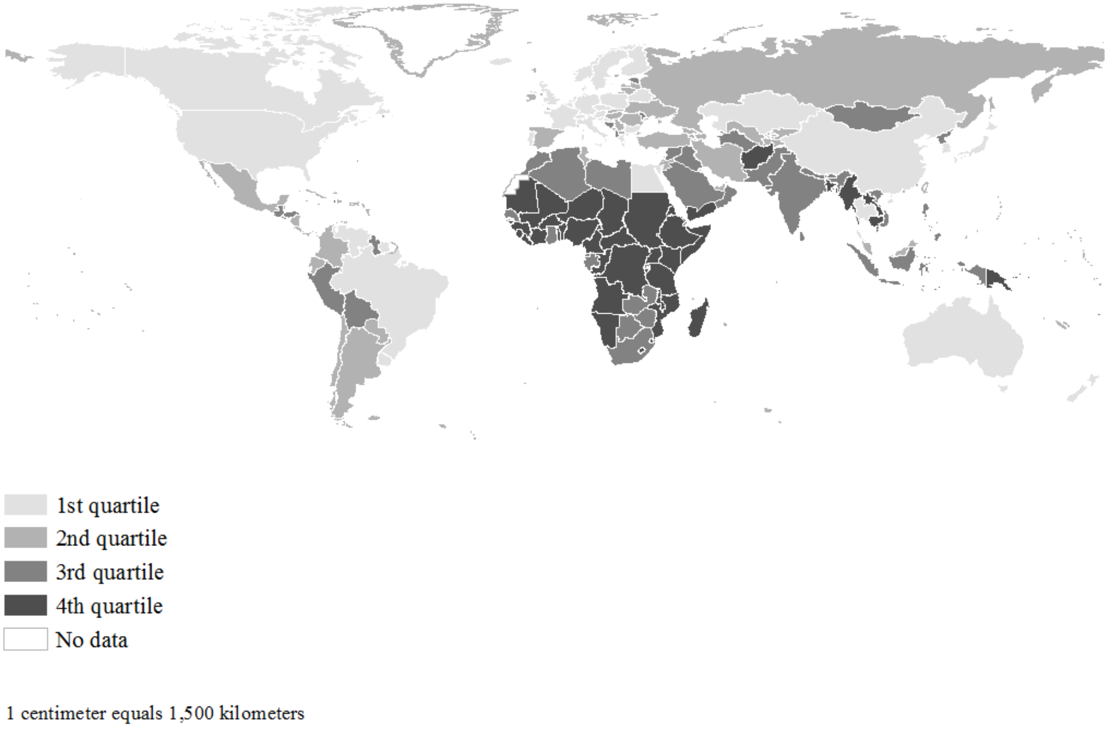

Figure S3 Climate adaptive capacity score, 2010. Colors Shading reflects climate vulnerability quartiles, with cut points of 0.36 (25th percentile), 0.48 (50th percentile), and 0.65 (75th percentile). Mean score was 0.51, with a range of 0.79 (min=0.15; max=0.94). Data taken from the Country Index of the Notre Dame Global Adaptation Index (ND-GAIN), and cover 179 countries. Data deficient countries shown in white.
